# Integrated Acetabular Prosthesis Versus Bone Grafting in Total Hip Arthroplasty for Crowe Type II and III Hip Dysplasia: A Retrospective Case–Control Study

**DOI:** 10.1111/os.14143

**Published:** 2024-07-23

**Authors:** Liangliang Cheng, Yuchen Liu, Linbao Wang, Jiawei Ying, Junlei Li, Fuyang Wang, Xing Qiu, Tianwei Zhang, Zhijie Ma, Yu Zhang, Bin Wu, Linpeng Liu, Liqun Song, Pinqiao Yi, Haiyao Wang, Dewei Zhao

**Affiliations:** ^1^ Department of Orthopedics Affiliated Zhongshan Hospital of Dalian University Dalian China; ^2^ Laboratory of Orthopedics Affiliated Zhongshan Hospital of Dalian University Dalian China

**Keywords:** 3D‐printed, Autologous Bone Grafting, Developmental Dysplasia of the Hip, Integrated Acetabular Prosthesis, Total Hip Arthroplasty

## Abstract

**Objective:**

Many methods of acetabular reconstruction with total hip arthroplasty (THA) for Crowe type II and III adult developmental dysplasia of the hip (DDH) acetabular bone defect have been implemented clinically. However, there was no study comparing the results of integrated acetabular prosthesis (IAP) with bone grafting (BG). This study aims to investigate the efficacy of IAP and BG for acetabular reconstruction in Crowe type II and III DDH.

**Methods:**

The clinical data of 45 patients with unilateral Crowe type II and III DDH who underwent THA from January 2020 to January 2023 were retrospectively analyzed. The patients were divided into two groups: 25 patients using 3D‐printed IAP (IAP group) and 20 patients using BG (BG group). The operation time and intraoperative blood loss were recorded. The clinical outcomes were assessed by Harris Hip Score (HHS) and full weight‐bearing time. The radiological outcomes were evaluated by the radiological examination. Accordingly, intraoperative and postoperative complications were observed as well. The data between the two groups were compared by independent sample t‐tests and the Mann–Whitney U rank sum test.

**Results:**

There were no significant differences between the two groups in Harris Hip Score (HHS) (preoperative, 6 months postoperative, and the last follow‐up), leg length discrepancy (LLD), cup inclination, cup anteversion, vertical center of rotation (V‐COR), horizontal center of rotation (H‐COR) (*p* > 0.05). The mean HHS in the IAP group was higher than in the BG group at 1 and 3 months postoperative (*p* < 0.001). The mean surgical time and blood loss in the IAP group were less than in the BG group (*p* < 0.001). The mean full weight‐bearing time in the IAP group was shorter than in the BG group (*p* < 0.01). No complications were observed in either group during the follow‐up period.

**Conclusion:**

IAP and BG have similar radiographic outcomes and long‐term clinical efficacy in THA for Crowe type II and III DDH, but the IAP technique has higher surgical safety and facilitates the recovery of hip joint function, which is worthy of clinical promotion.

## Graphical abstract

To compare two methods of anatomical reconstruction of Crowe II and III DDH acetabulum (integrated acetabular prosthesis and bone grafting).

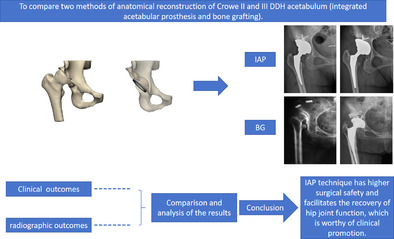

## Introduction

Developmental dysplasia of the hip (DDH) is characterized by abnormal acetabular and femoral anatomy.^1^ This anatomical anomaly leads to increased stress on the hip joint, causing it to be in a non‐physiological wear state for an extended period. Gradually, this results in anatomical and biomechanical abnormalities in the hip joint, hip joint instability, impingement and pathological changes in the acetabular labrum, ultimately progressing to degenerative arthritis.^2^ Despite some non‐surgical alternative treatments, many patients with late‐diagnosed DDH ultimately require total hip arthroplasty (THA) for treatment.^3^ According to Crowe et al.'s classification proposed in 1979, DDH is divided into I–IV types based on the degree of femoral head displacement.^4^ Patients with Crowe type II and III DDH often have a small and shallow acetabulum, and a false acetabulum forms above the true acetabulum after dislocation of the femoral head, with the overlap between the two prone to causing bone defects.^5^ In such cases, THA for Crowe type II and III DDH patients presents greater challenges than patients with ordinary osteoarthritis, with a risk of postoperative prosthetic loosening due to insufficient acetabular cup coverage.^6^ Therefore, for Crowe type II and III DDH undergoing THA, selecting an effective acetabular reconstruction method and ensuring sufficient acetabular bone coverage are crucial to guarantee the initial stability of the acetabular cup.

Crowe type II and III DDH with THA may choose to achieve sufficient bone coverage for acetabular cup stability by moving the hip joint center upward or inward.^7^ However, the movement of the rotational center may lead to changes in the biomechanics of the hip joint.^8^ To restore the rotational center of the hip joint and establish normal biomechanics, the reconstruction of the acetabulum in the true acetabulum position uses autologous bone grafting (BG) technique to provide structural support for the acetabular cup, bringing its position closer to the normal anatomical location, and at the same time providing sufficient bone mass for future revision.^9^ However, complications such as absorption and collapse of the bone grafts can easily lead to instability of the acetabular component.^10^ In recent years, with the increasing application of 3D technology in preoperative planning and intraoperative guidance, many difficulties in traditional orthopaedic surgery have been solved.^11^, ^12^ The 3D‐printed integrated acetabular prosthesis (IAP) technique can also be used to achieve acetabular reconstruction in THA for Crowe type II and III DDH.

In addition, the selection of appropriate prosthesis materials can improve the adaptability of 3D‐printed acetabular prosthesis, which is also the key to the success of Crowe type II and III DDH acetabular reconstruction surgery. Compared with traditional prosthetic materials, porous tantalum (P‐Ta) not only has extremely high biocompatibility, but also has biomechanical properties similar to human cancellous bone.^13^ It has been gradually used in the field of orthopaedics. Therefore, in this study, P‐Ta was selected as the material for preparing IAP due to its better bone integration ability and superior biomechanical performance.

This study collected and analyzed the clinical and radiological data for patients undergoing 3D‐printed IAP and BG surgical procedures for Crowe type II and III DDH. We aimed to (i) analyze and compare the clinical efficacy of 3D‐printing IAP and BG in the treatment of Crowe type III DDH and (ii) describe the surgical methods and skills of 3D‐printed IAP.

## Material and Methods

### Clinical Data

The study was a retrospective cohort study, which was conducted by searching the information system of the Affiliated Zhongshan Hospital of Dalian University from January 2020 to January 2023. A total of 45 patients with Crowe type II and III DDH were enrolled, including 13 males and 32 females, aged 45–72 years (average age: 58.2 years). The acetabulum was reconstructed with 3D printed IAP in 25 patients (IAP group) and BG in 20 patients (BG group). There was no significant difference in age, sex, BMI, Crowe classification, and follow‐up time between the two groups (*p* > 0.05, Table 1). The study was approved by the Ethics Committee of Affiliated Zhongshan Hospital of Dalian University (IEC‐C‐2021050‐1).

**TABLE 1:** Demographic of the study participants.

	IAP group	BG group	*t*/χ^2^/W value	*p* value
Number of patients	25	20	‐	‐
Age (years)	58.5 ± 7.4 (range, 45–70)	57.8 ± 7.1 (range, 50–72)	0.311	0.757
Sex			0.654	0.419
Female	19 (24.00%)	13 (35.00%)		
Male	6 (76.00%)	7 (65.00%)		
BMI (kg/m^2^)	22.5 ± 3.2 (range, 17.6–28.3)	22.8 ± 2.6 (range, 17.3–27.1)	0.453	0.866
Crowe classification			0.075	0.783
II	9 (36.00%)	8 (40.00%)		
III	16 (64.00%)	12 (60.00%)		
Follow‐up (months)	25.0 (16.5, 29.5)	30.0 (21.2, 31.8)	165.3	0.695

### Inclusion and Exclusion Criteria

Diagnosis of unilateral Crowe type II and III DDH patients had based on the criteria that the ratio of the distance from the head and neck junction to the interteardrop line to the femoral head diameter is between 50% and 100%, or the ratio of this vertical distance to the pelvic height is between 10% and 20%.^14^

Inclusion criteria: (i) cases undergoing primary THA; (ii) patients with severe hip joint dysfunction, pain, meeting the criteria for Crowe type II and III classification, and having osteoarthritis before joint replacement; (iii) surgery performed by the same senior physician.

Exclusion criteria: (i) patients aged less than 30 or more than 80 years; (ii) primary diseases of the hip joint are other autoimmune diseases, infectious arthritis, or tumor lesions; (iii) follow‐up period less than 1 year; (iv) lack of clinical records and radiographic images.

### Operation Procedure

**FIGURE 1:**
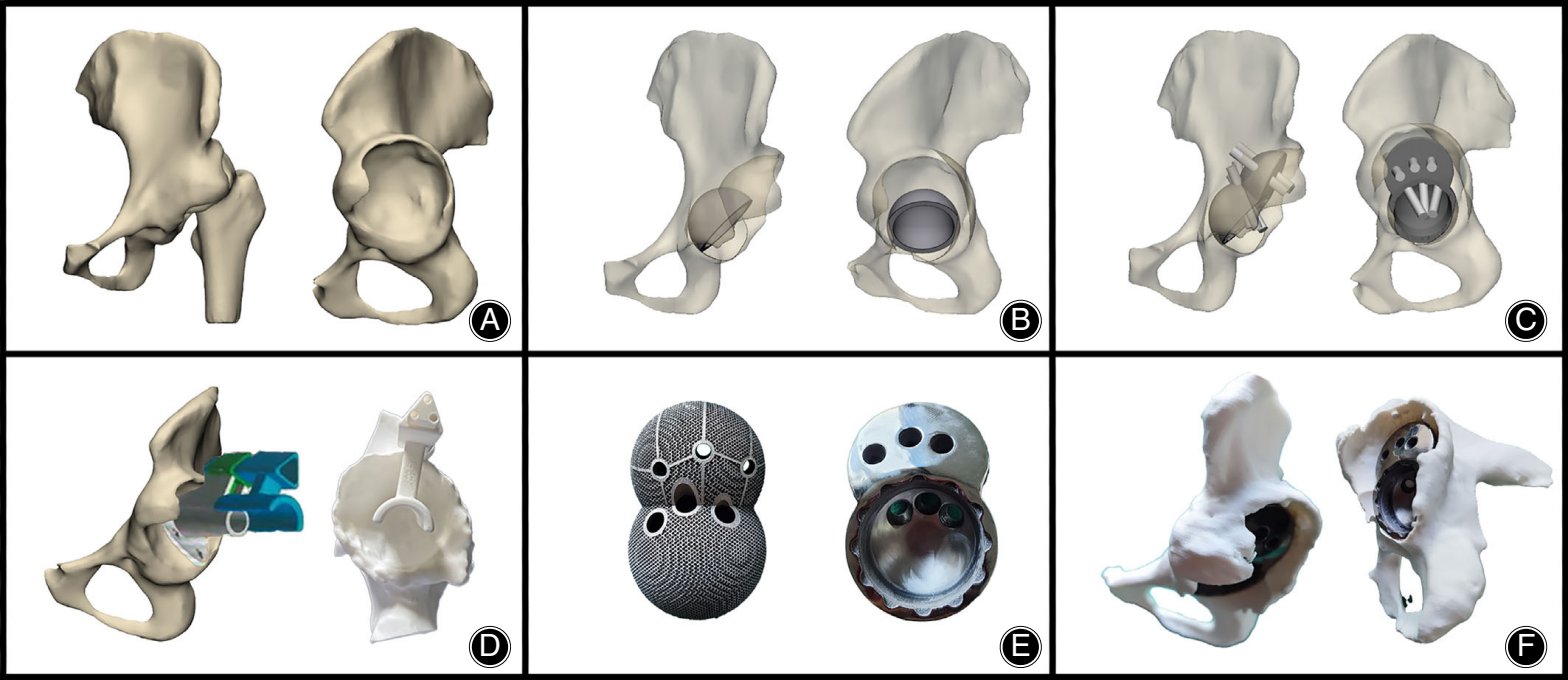
Preoperative prosthesis design. (A) Three‐dimensional reconstruction of the patient pelvic model. (B) Appropriate placement of the hemispherical acetabular cup in the true acetabulum. (C) Construction of the IAP model with design screws placement direction. (D) Design of 3D printing guide plate. (E) The object of IAP. (F) Simulation of the compatibility between the IAP and the pelvis.

Preoperatively, a 3D reconstruction of the pelvic model was conducted using the patient's hip joint CT data. Subsequently, an appropriate hemispherical acetabular cup was placed, and based on the acetabular defect in the model, a computer‐aided design system was utilized to construct a model of the IAP, determine the length of the screws to be used, and ascertain the placement direction. Meanwhile, in order to ensure the absolute unity of preoperative design and intraoperative application, we also designed a 3D‐printed guide plate. The design concept is to create a model that is fully compatible with the patient's partial acetabular bone surface, and to mark the position and direction navigation that needs to be worn on its surface. In order to avoid stress shielding, the final design of the IAP model is porous through Magics (Materialize, Belgium). The established porous IAP model data is imported into a 3D printer to use laser powder bed deposition technology to use tantalum powder for prosthesis preparation. Finally, the 3D printing technology was used to prepare the actual IAP according to the model, and the IAP was placed on the pelvic model for testing, so as to fully prepare the preoperative plan. The whole process from design to application takes about 8–10 days (Figure 1).

After general anesthesia, all patients were placed in a lateral position. The hips were exposed with an anterolateral approach, then the femoral head was sawn off, the osteophyte and synovium around the joint and the soft tissue in the cotyloid notch of the true acetabulum were removed to fully expose the acetabulum. IAP Group, the 3D‐printing guide plate was used to determine the grinding position, and the acetabular reamer was used to grind the true and false acetabulum to the preoperative planned size. The acetabular test model was installed, and its position was adjusted to be satisfactory. The IAP was then implanted and tamped, followed by the fixation of the acetabular prosthesis using cancellous bone screws and the installation of the liner. Group B, depending on the size and location of the acetabular defect, trimmed the excised femoral head to match the bone defect site. Acetabular reamers of smaller size were used to prepare the bone bed for grafting, removing cartilage and sclerotic bone. Subsequently, two to three high‐purity magnesium screws were used to fix the bone graft, and acetabular reamers were used to treat the graft and host bone graft bed as a whole. Finally, the acetabular prosthesis and liner were installed. Depending on the degree of development and deformity of the proximal femur, we chose an appropriate femoral stem and femoral head prosthesis to relocate the hip joint. After the drainage was placed, we closed the incision.

### Perioperative Regimen

For all patients, positive motion exercises were initiated in bed after recovery from anesthesia. Prophylactic intravenous antibiotics were used within the first 24 hours postoperatively. Additionally, low‐molecular‐weight heparin (LMWH) and painkillers were systematically managed to prevent deep venous thrombosis (DVT) and relieve pain, respectively. The drainage tube was removed within 24 hours.^15^ Remove the surgical sutures based on the postoperative wound‐healing condition 14 days after surgery. Instruct patients to bear weight and ambulate early based on postoperative imaging examinations.

### Clinical and Radiographic Assessment

Patients were requested to complete clinical and radiological assessments at postoperatively and at 1 month, 3 months, 6 months, 1 year, and annually thereafter. Clinical and radiographic outcomes were evaluated by two attending orthopaedic surgeons.

Clinical details were recorded, and clinical follow‐up included operating time, blood loss, full weight‐bearing time, Harris Hip Score (HHS), and complications.^16^ Full weight‐bearing time was defined as the time when the patient was able to walk independently without a walking aid after surgery. The complications are recorded, including early‐onset and late‐onset complications during the period of perioperation and follow‐up. The early‐onset ones consist of infection, intraoperative fractures, DVT, pulmonary embolism, and nerve paralysis. Meanwhile, the late‐onset ones consist of postoperative dislocation, graft nonunion, graft collapse, liner wear, osteolysis, and aseptic loosening.

**FIGURE 2:**
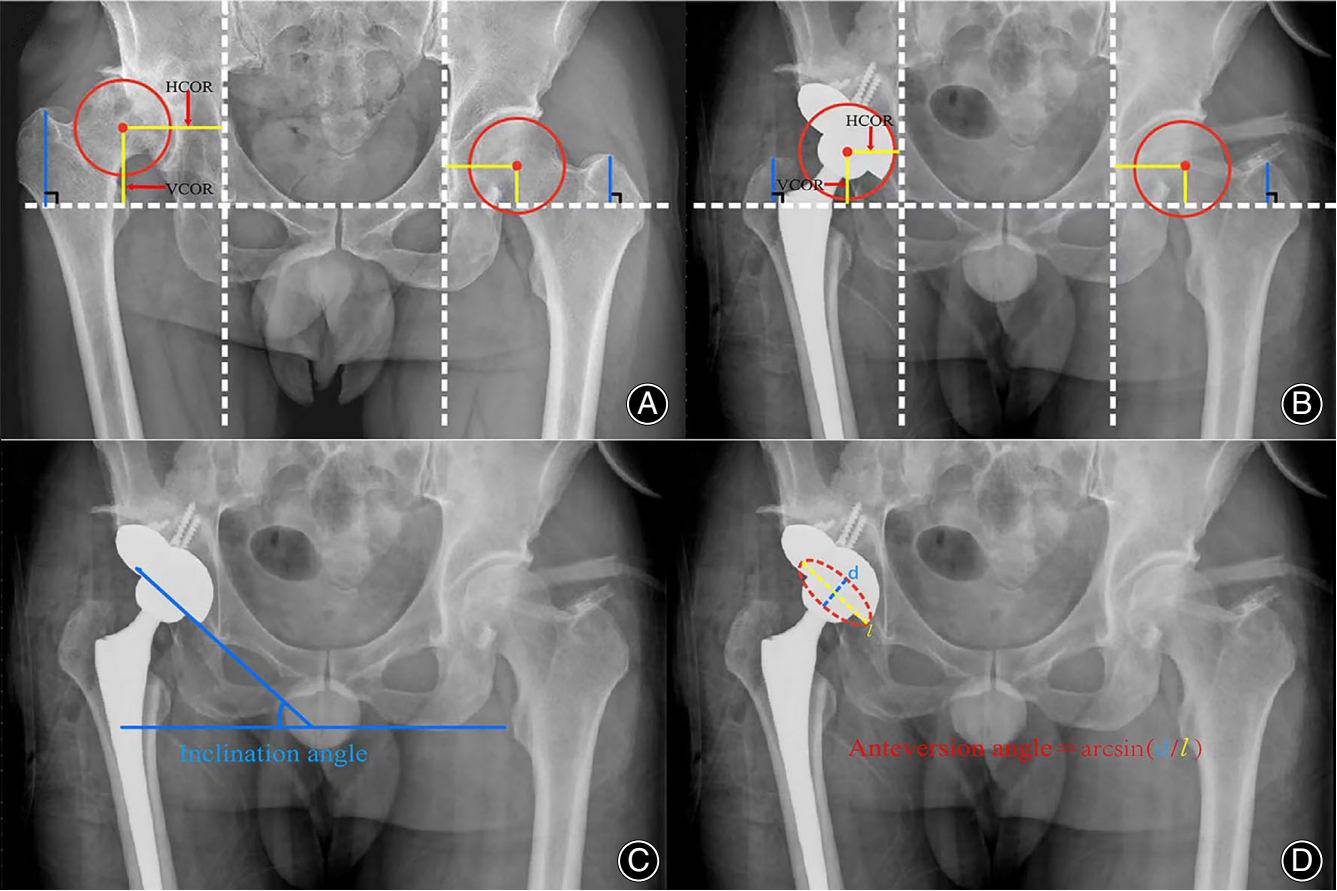
Radiographic assessments. (A) Preoperative V‐COR, H‐COR, and LLD measurements. (B) Postoperative V‐COR, H‐COR, and LLD measurements. (C) Cup inclination measurement. (D) Cup anteversion measurement.

Radiographic assessments included acetabular cup inclination, acetabular cup anteversion, leg length discrepancy (LLD), and position of the center of the hip joint. Measure the difference in vertical distance from the tip of the greater trochanter to the teardrop line on both sides to assess the LLD^17^ (Figure 2A,B). The hip center of rotation (COR) was evaluated by vertical center of rotation (V‐COR) and horizontal center of rotation (H‐COR). V‐COR was measured as the vertical distance between the center of the femoral head and the interteardrop line. H‐COR was measured as the horizontal distance between the center of the femoral head and the perpendicular line through the inferior point of the teardrop (Figure 2A,B).^18^ Cup inclination (°) was defined as the abduction angle between the interteardrop line and the tangent to the cup (Figure 2C). Cup anteversion (°) was measured using the method of Lewinnek et al.^19^ (Figure 2D).

### Statistical Analysis

Statistical analysis was performed using SPSS 20.0 software (IBM, New York, USA). In descriptive statistics, count data were expressed as numbers and percentages, and the chi‐square test or Fisher's exact test was used for comparison between groups. The measurement data conforming to the normal distribution were expressed as mean and SD, and the independent sample t‐test was used for comparison between groups. Measurement data with non‐normal distribution were expressed as median (interquartile range), and Mann–Whitney U rank sum test was used for comparison between groups. *p* < 0.05 was considered statistically significant.

## Results

**FIGURE 3:**
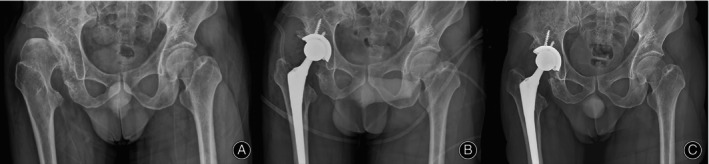
Radiographs of a 55‐year‐old man with right Crowe type III DDH underwent THA with BG technique. (A) Preoperation. (B) Immediate radiograph after THA showed complete coverage of the acetabular prosthesis with bone graft. (C) The radiograph at 3 years postoperatively showed similar bone density between the bone graft and the acetabulum bone, proper prosthesis position, and no prosthesis of loosening or sinking.

**FIGURE 4:**
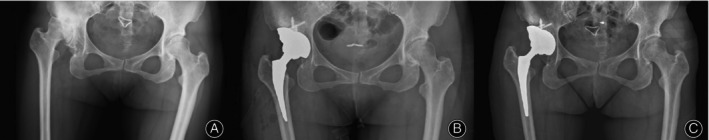
Radiographs of a 51‐year‐old woman with right Crowe type III DDH underwent THA with IAP technique. (A) Preoperation. (B) Immediate radiograph after THA showed good compatibility between the prosthesis and the host bone. (C) The radiograph at 2 years postoperatively showed stable acetabular prosthesis, and the anterior and posterior walls of the acetabulum were firmly supported by the IAP.

All 45 patients were followed up for 12–36 months, with an average of 26.9 months, and all patients completed the follow‐up successfully. Typical cases are shown in Figures 3 and 4.

### Comparison of Clinical Outcomes

The average HHS of the two groups was significantly improved after the operation. There was no significant difference in the average HHS score between the two groups before operation, 6 months after operation, and at the last follow‐up (*p* > 0.05, Table 2). The average HHS scores of the IAP group were higher than those of the BG group at 1 month and 3 months after operation, and the difference was statistically significant (*p* < 0.001, Table 2). The average surgical time and blood loss in IAP group was less than in BG group (*p* < 0.001, Table 2). The average full weight‐bearing time in IAP group was shorter than in BG group (*p* < 0.01, Table 2).

**TABLE 2:** Comparison of clinical outcomes of the patients in both groups.

	IAP group	BG group	*t* value	*p* value
Intraoperative blood loss (mL )	135.0 ± 39.3	224.3 ± 38.2	7.663	<0.001
Operative time (min)	61.8 ± 7.6	89.8 ± 6.9	12.690	<0.001
Time to full weight‐bearing (weeks)	5.2 ± 1.5	8.2 ± 2.1	4.409	0.002
HHS (points)				
Preoperative	48.3 ± 3.7	46.6 ± 3.8	1.519	0.136
1 month postoperative	75.2 ± 2.0	63.3 ± 3.1	14.680	<0.001
3 months postoperative	87.6 ± 3.1	80.2 ± 2.2	4.017	<0.001
6 months postoperative	90.3 ± 3.9	89.0 ± 5.3	0.958	0.353
Last follow‐up	91.2 ± 3.8	90.1 ± 4.6	0.880	0.383

### Comparison of Radiological Outcomes

The average LLD, V‐COR, and H‐COR after operation were significantly improved compared with those before operation, and the difference was statistically significant (*p* < 0.001, Table 3). There was no significant difference in the average acetabular cup inclination, acetabular cup anteversion, LLD, V‐COR, and H‐COR between the two groups (*p* > 0.05, Table 3).

**TABLE 3:** Comparison of radiographic outcomes of the patients in both groups.

	IAP group	BG group	t value	*p* value
Acetabular cup inclination (°)	42.3 ± 5.1	40.7 ± 3.6	0.774	0.443
Acetabular cup anteversion (°)	15.8 ± 2.7	16.2 ± 4.1	0.312	0.764
V‐COR (mm)				
Preoperative	42.3 ± 5.3	43.3 ± 6.0	0.250	0.804
Postoperative	18.1 ± 2.8	19.3 ± 3.1	1.339	0.188
*t* value	20.390	16.750		
*p* value	<0.001	<0.001		
H‐COR (mm)				
Preoperative	44.4 ± 5.2	43.3 ± 4.6	0.710	0.481
Postoperative	29.5 ± 3.6	28.8 ± 4.1	0.641	0.524
*t* value	10.620	12.110		
*p* value	<0.001	<0.001		
LLD (mm)				
Preoperative	23.6 ± 7.4	24.2 ± 6.9	0.254	0.800
Postoperative	3.0 ± 2.1	3.1 ± 2.7	0.213	0.832
*t* value	14.360	13.080		
*p* value	<0.001	<0.001		

### Complications

There were no early complications in both groups. During the follow‐up period, there were no late complications such as dislocation, bone resorption, and translucent lines in both groups. In addition, we did not observe graft collapse or graft displacement.

## Discussion

This study confirmed that both the IAP group and BG group can achieve good clinical outcomes and improve the quality of life of patients. Although the preoperative design steps are added, the IAP group has the advantages of rapid recovery of hip joint function and higher surgical safety.

### Advantages of the True Acetabulum Position Reconstruction

Reconstruction of the true acetabulum is the primary approach for acetabular reconstruction in Crowe type II and III patients.^20^ This technique not only restores the anatomical rotation center of the acetabulum and ensures biomechanical stability, but also effectively mitigates postoperative LLD, improving gait and function.^21^

The ideal COR, V‐COR is about 14–20 mm, and H‐COR is about 15–38 mm.^22^ In this study, both methods of true acetabulum reconstruction showed no instances of prosthetic loosening or adverse events during follow‐up. This may be because the VCOR and HCOR of the two groups of patients returned to the ideal range, basically restoring the biomechanical characteristics of the normal hip joint. Additionally, restoring LLD not only reduces the symptoms of low back pain and claudication caused by pelvic and spinal tilt, improves the satisfaction of surgery, but also reduces the risk of aseptic loosening of the prosthesis, prolongs the service life of the prosthesis, and has significant benefits for patients.^23^ According to Nossa et al.,^24^ postoperative LLD is acceptable in the range of 0–1cm, and will not adversely affect the patient's subjective feelings and clinical function recovery. In this study, the final follow‐up showed that the LLD in both groups was less than 1 cm, with normal gait and no perceived LLD.

The COR and the LLD of the patients in the two groups of reconstruction methods were basically restored to the ideal state, indicating that our two acetabular reconstruction techniques have achieved satisfactory anatomical reconstruction of the acetabulum.

### Challenges Faced by the BG Technique

**FIGURE 5:**
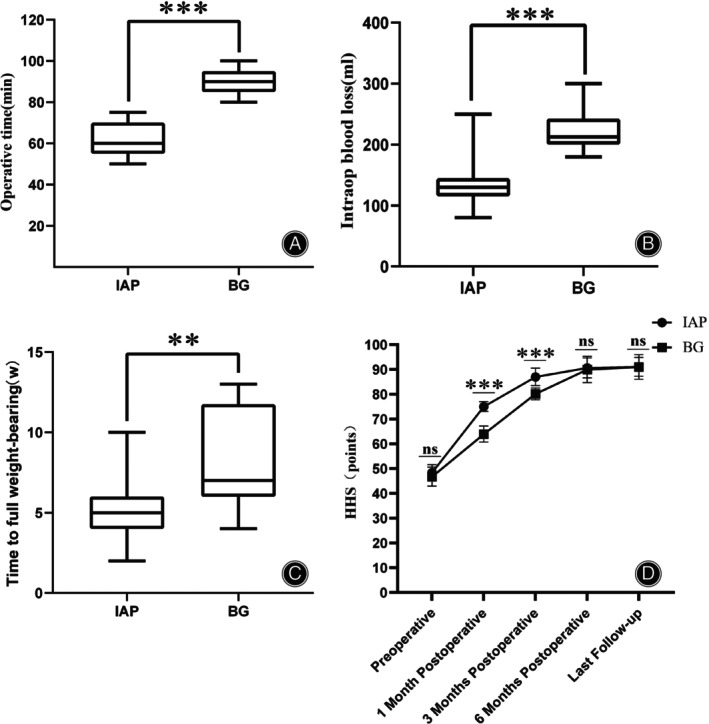
Comparison of clinical data of the patients in both groups. (A) Comparison of operative time. (B) Comparison of intraoperative blood loss. (C) Comparison of time to full weight‐bearing. (D) Comparison of dynamic HHS.

The BG technique is a good choice for the reconstruction of Crowe type II and III DDH acetabulum. Using metal screws to fix the bone graft and reconstruct the acetabulum in the real acetabulum position, while ensuring the stability of the acetabulum cup, it also increases the bone reserve, so that the acetabulum cup prosthesis bone ingrowth reaches biological fixation.^25^ However, the BG technique also faces many problems. First of all, titanium alloy screws are often used to fix the traditional structural bone graft bone block above the outside of the acetabulum. Due to the large difference between its elastic modulus and bone tissue, there is often stress shielding.^26^ Some scholars have developed high‐purity magnesium internal fixators with similar elastic modulus to bone, which have shown stable mechanical properties and good biocompatibility in clinical applications.^27^, ^28^ Even so, the BG technique requires time for bone graft healing. Early full weight‐bearing may increase the risk of bone graft collapse and nonunion.^29^ Patients cannot get off the ground as soon as possible after surgery, and the recovery time is longer.^30^ In addition, the BG technique also requires intraoperative trimming of bone blocks, which prolongs the operation time. At the same time, the prolongation of the operation time causes more bleeding in the bone wound, and more trauma and bleeding in the bone extraction area are also the reasons for more bleeding in the BG technique (Figure 5). These results increase the risk of surgery.

### The Advantages of 3D‐Printed IAP

In recent years, with the application of 3D‐printed technology in orthopaedic surgery, personalized precision treatment provides a new idea for the reconstruction of acetabular bone defects.^31^ First of all, we used 3D‐printed technology to design an IAP that meets the size and shape of Crowe type II and III DDH acetabular bone defects, which solved the problems of insufficient bone mass and unsatisfactory shape of the BG technique. The 3D‐printed IAP achieves a perfect match with the patient's acetabular bone and can better coordinate with human biomechanics. Secondly, the integrated structure can not only avoid the risk of fracture and loosening of multi‐component prosthesis, but also reduce the complexity of the surgical operation and improve the safety of surgery.^32^ Finally, 3D‐printed technology can realize the design and manufacture of porous bionic trabecular bone structures.^33^ The IAP of porous structure is closely combined with bone tissue and obtains immediate stability. Then, through the growth and ossification of trabecular bone, the bone tissue grows into the micropores on the surface of the prosthesis, realizing the transformation from mechanical fixation to biological fixation, so that IAP can obtain satisfactory biomechanical stability. Patients can start early full weight‐bearing, which is conducive to the recovery of hip function.

### Limitations

As an exploratory trial to compare the efficacy of two acetabular reconstruction methods, this study also has some inevitable limitations. Firstly, the number of cases in this study is small, and there is no long‐term follow‐up, so the long‐term clinical effect needs to be further observed. Secondly, this study is a retrospective summary, which is subject to selection bias, and evidence from prospective, randomized, controlled studies is still needed.

## Conclusion

The application of 3D‐printed IAP in the reconstruction of the acetabulum for Crowe type II and III DDH patients exhibits radiological outcomes and long‐term clinical efficacy consistent with the BG. Moreover, it offers higher surgical safety and faster functional recovery of the hip joint after surgery. This approach holds promising prospects for reliable clinical application in the reconstruction of Crowe type II and III DDH acetabulum.

## Conflict of Interest Statement

The researcher claims no conflicts of interests.

## Author Contributions

Conceptualization and methodology: DZ, LC, YL; design and 3D printing: BW, LL, LS, PY, HW; performing the surgery: XQ, TZ, ZM, YZ; data collection, analysis, and interpretation: YL, LW; manuscript writing: LC, YL, LW; revision of manuscript: JL, FW, JY; supervision: DZ. LC and YL contributed equally to this work and should be considered as equal first authors.
